# Partnership between Primary Health and Social Care Services in the Long-Term Care of Older People with Dementia: A Vignette Study

**DOI:** 10.1177/00469580211011933

**Published:** 2021-04-23

**Authors:** Ausrine Kontrimiene, Aurelija Blazeviciene, Ida Liseckiene, Gediminas Raila, Leonas Valius, Lina Jaruseviciene

**Affiliations:** 1 Lithuanian University of Health Sciences, Kaunas, Lithuania

**Keywords:** primary health care, mental health services, social work, long-term care, patient care, health personnel, dementia, vignette study, qualitative research

## Abstract

Dementia is considered to be a significant cause of disability and dependency for older people worldwide and it raises difficulties in providing adequate formal and informal assistance. Research on the experience of long-term care (LTC)services for older people with dementia is scarce in Eastern European countries. This study aimed to understand the system of care for older people with dementia from the perspective of health and social care workers providing LTC services in Lithuania. A total of 72 primary health care and social care professionals from public and private institutions in Kaunas city participated in this study. One-to-one interviews were conducted with family physicians, community nurses, psychiatrists, psychiatric nurses, and social workers. A vignette situation of 2 fictitious patients with dementia and their informal caregiver was discussed during the interviews. Data were analyzed using thematic analysis by induction approach. The data revealed 2 main themes: LTC provision trajectory, and three-dimensional relationship perception in realization of LTC activities. LTC provision trajectory reflected activities performed as a response to the described situation embracing formal procedures for the endorsement of LTC needs as well as the range of LTC services. The three-dimensional perception of relationships in LTC services’ implementation reflected the participants’ personal approach toward LTC, relationship with different specialists, and the informal caregiver. Our study revealed the potential of complex measures that could be instrumental for the refinement of the caregiving process. First, a change in the additional care requirements endorsement logic is needed, shifting focus from medical diagnosis to functional abilities assessment. Second, to establish clear procedures for formal cooperation between the health and social care sectors in the trajectory of LTC service provision. Finally, to find an adequate balance between LTC and institutional care by creating a more comprehensive range of LTC services. A more consistent and coordinated delivery of services by both health and social care sectors seems to be an untapped resource for the improvement of the LTC potential.

**What do we already know about this topic?**
Dementia is considered to be a significant cause of disability and dependency for older people worldwide and it raises difficulties in providing adequate formal and informal assistance.
**How does your research contribute to the field?**
Our study revealed the potential of complex measures that could be instrumental for the refinement of the caregiving process in Central and Eastern Europe, such as: to establish clear procedures for formal cooperation between the health and social care sectors in the trajectory of LTC service provision; to implement a more consistent and coordinated delivery of services by both health and social care sectors; to find an adequate balance between LTC and institutional care by creating a more comprehensive range of LTC services.
**What are your research’s implications toward theory, practice, or policy?**
We aim to shed more light on the collaboration challenges between health and social care sectors in the Lithuanian region. Furthermore, to reveal the issues relevant to Eastern and Central Europe.

## Background

Aging population dramatically challenges the socio-economic, health, and social care areas of the societies of middle- and high-income countries.^1^ Multiple chronic conditions, including dementia are present in approximately 80% of older adults creating increasing pressure on care systems worldwide.^2^ Dementia is seen as a significant cause of disability and dependency in older people, which also has a profound impact on the lives of their families and communities.^3,4^ It is estimated that the number of people living with dementia worldwide is currently over 50 million and may increase up to 152 million by 205.^5^ This creates a significant economic burden of dementia care on patients, their informal caregivers, and healthcare systems.^6^ However, it is nursing and informal care, and not the direct medical costs, that contribute the most to the total care costs of patients with dementia (e.g., home-based long-term care (LTC), nursing homes).^4,6^ Furthermore, it negatively affects the work efficiency and employability of the informal caregivers in the labor market.^4,6^

Research suggests that patients with complex LTC needs experience multiple parallel care processes, which may have conflicting or competing goals within their individual patient trajectories.^7^ Thus, the reorganization of social and health care processes focusing on dementia patients has become one of the top priorities across countries, and a large array of tools, including attitudinal, organizational, and regulatory transformation of care delivery, have been applied with the aim to foster integrated service delivery (i.e., promoting interprofessional teamwork, continuing education programs for health and social care professionals, a common information system for both sectors, results-oriented learning, service users’ involvement in developing and implementing integrated services).^3,7-10^ Studies indicate that a partnership between healthcare and social services results in higher user satisfaction with the care received,^11,12^ and lower avoidable healthcare use and spending.^13^ However, different collaborative actions and frameworks of integrated care are being discussed to meet the needs of country-specific health and social care systems, taking into account the cultural and national aspects of the systems.^14-16^

Fragmentation of health and social care is also an issue in Lithuania where there is a lack of collaboration between family physicians and social workers.^10^ It is especially problematic in the financial situation when social security expenditures per person in Lithuania reach almost half of the EU average.^12^ Moreover, research has also shown that the level of cooperation in the Lithuanian primary health care (PHC) sector in the care of patients with mental health problems was insufficient.^10^

Previous legislative attempts to foster collaboration between these sectors, such as funding of joint activities, had a low impact on service provision and left organizational frameworks unchanged.^17,18^ Lack of collaboration is especially detrimental in the context of financial strain—spending on social protection per person in Lithuania barely reaches half of the EU average;^19^ the health expenditure in the financial year 2017 accounted for 6.5% of the GDP, which was substantially lower than the EU average of 9.8%.^20^ Health care spending on LTC is 7.9% below the EU average.^20^ The demand for both institutional and home care provision widely exceeds the supply,^21^ and as a consequence, the number of avoidable hospitalizations is high.^22^ The financial expression of these circumstances was illustratively reflected in a previous research comparing the cost of dementia in different regions of the world: the direct social cost of dementia in Eastern Europe (EE) is relatively low (20.7%)as compared to the global mean (40.1%); however, health care and informal care are comparatively overloaded (direct health care costs in EE were 24.1% compared to the global mean of 19.5%; informal care costs in EE were 55.2% compared to the global mean of 40.4%).^23^

Lack of partnership and coordination of care activities between health and social care sectors could negatively affect the well-being and safety of older patients with dementia and impose an additional burden on their informal caregivers.^24,25^ Various efforts have been made to address the Lithuanian situation of care for older patients with complex needs including dementia, mainly focusing on the experience of informal caregivers,^26,27^ piloting the integrated care delivery project.^28,29^ The integrated care project’s idea was to complement existing social care services with newly created teams of nurses, their assistants, and physiotherapists employing them in the social care sector. This innovation was successful and funded by the European Social Fund, but its sustainability was challenged when external funding ended.^28,29^ However, research on long term care for older patients with complex needs is missing not only in Lithuania, but also in other Eastern European countries.^21,30^ Therefore, this study aimed to bridge this research gap, assessing the partnership between the formal caregivers from health care and social sectors providing LTC services for the older patients with dementia in Lithuania.

## Methods

This study is a part of a larger project titled “*Integrated Health Care for Senior’s Mental Health: Developing an Intersectoral Cooperative Care Model.”* The 3-year project (2017-2020) was financed by the Lithuanian Research Council (S-MIP-17-121). The aim of the project was to develop a better understanding of the primary healthcare and social care collaboration and management of older people with dementia and their informal caregivers, and to find pathways for improvement in care. The views regarding the care system held by the informal caregivers attending to elderly patients with mental disorders have been discussed elsewhere.^26^ The focus of this study was to understand the system of care for older patients with dementia in Lithuania from the perspectives of family physicians (FPs), community nurses (CNs), psychiatrists (PPs), psychiatric nurses (PNs), and social workers (SWs).

All of the researchers participating in the study were specially trained in working with qualitative research. Almost all of them were family physicians and employees of the Lithuanian University of Health Sciences. One was from the nursing department of the Lithuanian University of Health Sciences. One of the researchers had a master’s degree in Applied Sociology.

The Regional Committee on Biomedical Research Ethics of Kaunas approved this study on 2018-04-23 (No: BE-2-47).

### Context of Lithuanian Health Care and Social Care Systems

Municipalities organize the provision of primary health care and social services in Lithuania. Lithuanian primary health care consists of public and private primary healthcare centers, which work under an agreement with the National Sickness Fund (NSF) and provide free services to the patients. The leading PHC providers are family physicians and community nurses, and the leading providers of primary psychiatric care are psychiatrists, psychiatric nurses, social workers, and psychologists.

Social care in Lithuania traditionally was dominated by institutional care, development of home care services started within the last few decades. Home care services are provided by municipal state social care institutions and private, non-governmental institutions that provide services when the need for services exceeds public institutions’ capacity. Providers of social care services at patients’ homes are social workers and social work assistants.

The lack of cross-sectoral cooperation is not limited to different organizational structures.^26^ Health care services are free of charge, while social care services always require co-payment. Primary health care services are provided in every case when there is a need. In contrast, there are several mandatory conditions for receiving social services at home—that is, older patients must have formally established additional care requirements to receive any home care services. The whole health care is coordinated by family physicians serving both as health system gatekeepers and gate-openers. Social services provision mainly remains specialized without coordination between the sectors and within the sector. Additional care requirements are among the essential requirements to receive home care services. The formal establishment of additional care requirements is performed by Disability and Working Capacity Assessment Office under the Ministry of Social Security and Labor. It is based on GPs referral justifying patients’ medical condition (e.g., diseases, their severity, diseases’ effects on looking after yourself). There are 2 levels of patients’ additional care requirements—constant care and constant nursing, both followed by a different number of financial benefits, reimbursement of medicines, technical assistance devices and the provision of health and social care services including LTC.

### Study Design

A vignette study method was chosen to get a deeper understanding of the viewpoint of the different professionals involved in the care of the older people with dementia. Our group of researchers prepared the vignette as a brief, carefully written description of a situation designed to simulate key features of a real-world scenario regarding dementia care. Methodologically, we decided to include controlled aspects of the vignette consistent during the interviews to avoid extraneous differences between all professions. The theoretical framework of the vignette investigation had 2 main elements: professional practice experience at the micro level (i.e., formal procedures, range of functions, and challenges in formal caregiving) and intersectoral collaboration trajectory (i.e., pathways of partnership between sectors and challenges of mutual collaboration).We chose the vignette method as it allows for better insights into the micro level of health and social care systems and reveals the personal experiences of professionals engaged in the processes of care.

A vignette situation involving 2 fictitious patients (spouses) with dementia and their daughter was created by our group of researchers based on a previous research with informal caregivers^26^ and the opinions of experts. The vignette was piloted with a group of experts, which included all the professions to be included later in the study, that is, family physicians, community nurses, psychiatrists, psychiatric nurses, and social workers. Revisions were made to the vignette based on their feedback. The final version of the vignette was approved by both the experts and the researchers. Five open-ended questions to be presented following the situation were prepared for the participants (Table 1). All participants signed informed consent forms and were informed about the course of the study. Participants were provided with information on confidentiality and the opportunity to withdraw from the study at any time.

**Table 1.** Questions for the Vignette Situation.

1. What would you identify as the main problems in this situation?
2. How do you usually find out about this situation?
3. How would you deal with this case: what would you do during this visit?
4. How would you deal with this case: what would be your long-term care plan?
5. What is your experience with other professionals in dealing with such situations?

### The Vignette

Spouses Anthony (85 years old) and Amelia (84 years old) live in a 2-bedroom apartment. The patient’s daughter, who lives in another apartment, also participates in the visit. Anthony has no significant complaints about his health, except for a chronic ulcer in the calf area for the past several months. Amelia complains that the husband does not feed her and steals her money. You notice that the carpets seem to have been cleaned a while ago and the surfaces at home were dusty. Various medicine boxes are scattered on the kitchen table. The daughter says it has become increasingly difficult to help parents in recent years: they are reluctant to accept help but no longer manage their homes, do not take medication regularly. It is difficult for her to keep in touch with them because they do not answer the phone. The daughter tells you that Amelia no longer remembers how to cook and does not even remember that she has to eat, sometimes gets lost between the rooms. Amelia was diagnosed with vascular dementia, and additional care requirements of constant care were established 2 years ago. Anthony takes care of her: he dresses her up, gives her food and bathes her. The daughter is worried that Anthony’s memory is deteriorating as well. His behavior is changing (he forgets to eat, sometimes it’s hard for him to make a simple dish (e.g., making sandwiches)). The daughter says she wants to help her parents but does not know how to do it.

### Data Collection

The study was conducted in Kaunas, which is a highly urbanized central city in Lithuania. The data was collected during the spring and summer of 2019. Primary health care professionals from public and private clinics of Kaunas city were invited to participate in the study. Snowball sampling was used to invite professionals from different practices who were willing to participate in the study.

Four researchers specially trained in qualitative data gathering conducted the interviews. The interviews were audiotaped or handwritten. Handwritten data was written shorthand during the interviews. After each interview, the researcher expanded the collected data into sentences and prepared the final version of the interview for the analysis. Audiotaped interviews were transcribed verbatim.

Data collection and initial data analysis proceeded in a linear process. We reviewed all the data collected in each group of study participants separately. When no new codes were identified during the interviews of a particular group of participants, we conducted 2 additional interviews. If no new information was found, we considered that the saturation was reached for that particular group and stopped the data collection. If new information was found, we continued the interviews until 3 consecutive interviews identified no new information.

### Data Analysis

Data were analyzed using thematic analysis by induction approach.^31^ The thematic analysis was initiated after all interviews in each group of the participants were completed. The codes were generated systematically for the entire dataset by reviewing the data line by line. Two researchers, specially trained to perform qualitative data analysis, performed the coding independently. The terms chosen for coding were as similar as possible to the participants “own choice of words.” Upon completion of the initial coding, the 2 coded transcripts were systematically compared, and approximately 90% of the codes in both coded datasets were similar. The remaining codes were discussed between the 2 researchers until a joint decision was reached about coding. Related codes were grouped into thematic categories. The final themes were formulated according to the grouping of thematic categories. The whole dataset with grouped categories and themes were reviewed and specified. Verbatim extracts of the participants’ interview data have been used to illustrate the categories. The source of each illustration has been labeled at the end of the quote (e.g., “CN2” denotes the number of an interview with a community nurse), omitted sentences have been marked with bracketed ellipses [. . .], and researchers’ comments have been given in brackets (e.g., [family physician]).

## Results

### Participants

A total of 72 participants took part in the study (Table 2), of which 68 were women. Mean age of the participants was 48 years (SD = 11.65). The participants worked at private (n = 24) and public (n = 48) primary health care centers. The sociodemographic data of the participants are presented in Table 2.

**Table 2.** Sociodemographic Data of the Participants.

Indicator	n	%
Gender		
Female	68	94.4
Male	4	5.6
Mean age (years)	48 (SD 11.65)	
	Min 22; max 70	
	IQR 18.75	
Profession		
Family physician	19	26.4
Community nurse	18	25.0
Psychiatrist	13	18.1
Social worker	12	16.7
Psychiatric nurse	10	13.9
Type of practice		
Public	48	66.7
Private	24	33.3
Total number of participants	72	100.0

SD = standard deviation; IQR = interquartile range.

### Themes and Categories

The data revealed 2 main themes with categories within each theme (Table 3). LTC provision trajectory reflected activities performed as a response to the described situation embracing formal procedures for the endorsement of LTC needs and the range of LTC services. The three-dimensional perception of relationships in LTC services’ implementation reflected the participants’ personal approach toward LTC, relationship with different specialists and the informal caregiver.

**Table 3.** Themes and Categories that Emerge from the Thematic Data Analysis.

Themes	Categories
Long-term care provision trajectory	Formal procedures for endorsement of LTC needs
	Range of LTC services
Three-dimensional relationship perception in realization of LTC activities	Relationships between health and social care providers
	Relationships with informal caregivers
	Personal approach to LTC provision

### Long-Term Care Provision Trajectory

#### Formal procedures for endorsement of LTC needs

The study participants of all professional profiles emphasized the need to formally confirm the additional care requirements of both patients, a necessary bureaucratic formality to obtain LTC services. Formal (bureaucratic) identification of additional care requirements is a launching mechanism of LTC services both for nursing and other social care services: “*We wouldn’t get to these people because they don’t have additional care requirements for nursing[. . .] we only go when they have additional care requirements for nursing[. . .],” SW3.* However, the participants expressed that the establishment of this formal (bureaucratic) requirement has exceptions in the health care sector and depends on the medical criteria. The participants expressed doubts about whether the status of both the patients presented in the vignette could qualify the formal criteria for additional care requirements endorsement for nursing.

*It is difficult for them to leave the house. Although, it is unlikely that they would be identified with additional care requirements for nursing because they are not bedridden. We have situations in the visiting care where the [functional] condition of people is very poor and additional care requirements for nursing are not established [. . .], SW3*

#### Range of LTC services

The participants’ experiences indicate that the formal endorsement of additional care requirements unlocks the LTC possibilities realized by health and social care providers. The spectrum of services provided at the patient’s home ranges from education of informal caregivers (performed by GPs or CNs), to assistance in medication management (performed by CNs), bedsore treatment (performed by CNs), and general household assistance (performed by CNs or SWs) with the involvement of municipal bodies and non-profit organizations. Institutional nursing, in contrast to home care, seems to be accessible to all patients with and without additional care requirements’ endorsement. This possibility was repeatedly mentioned by all the participants as the only tangible long or short-term solution to the situation: “*I would recommend inpatient treatment in a nursing hospital until a further decision is made on how to organize patient care,” CN16.*

### Three-Dimensional Relationship Perception in Realization of LTC Activities

#### Relationships between health and social care providers

The participants’ insights revealed that cooperation between health and social care providers is weak and abstruse. Both health and social care providers admitted facing challenges for partnership, ranging from difficulties in information sharing about the particular needs of the patient to a more general non-cooperative attitude between the sectors as there is no routine feedback regarding the patient care needs from either side.

*Facing organizational aspects – workers do not answer, are on holidays or business trips or on sick leaves and there is no one to replace them, you leave your contact information, but no one calls back. I ask for feedback, but it’s not there. I pass on the information and you don’t know what’s going on, you don’t get answers. There is no cooperation with the social services department, no feedback, SW1.*

When there is no communication, there are no problems. We don’t have a social worker, so we don’t even know what they can do. We try to solve the problems together with the informal caregivers, FP3.

The following circumstances, unfolded by the participants, may underlie this non-cooperative attitude:

- Health and social care sectors are 2 different cultural systems fairly uninformed of each other. The participants revealed discrepancies in professional goals of health and social care providers and a lack of experience in arriving at common priorities.

*They [physicians] don’t have time and you feel underappreciated for the work. You try to be a professional in your field, but there is an imposition of opinion, they seem to know everything, overestimated self-esteem. Lack of cooperation, communication, different priorities, high workloads, SW1.*

- It appears that health care providers lack the knowledge of the social care sector structure and are poorly informed about social workers’ roles and functions. Additionally, the providers from each sector lack contacts in the other sector and do not know where to look for them.

*I tried many times to look for [a social worker] but failed. I also searched the Internet and called. They don’t have enough staff and the waiting list is long. I called private ones directly. There is no feedback from public ones, FP7.*

- Another aspect affecting potential cooperation between the professionals from the 2 sectors is attitudinal: unfriendliness and unwillingness to cooperate.

*The attitude of the medics is that we are better than you. This status comes across strongly, not mildly. As if we are the lowest link. I don’t feel that way. Maybe the older generation feels that way. I really don’t feel inferior and interact with doctors as I do with those on my own level, but [. . .] when I worked in the visiting service, it really felt bad, SW4.*

*Doctors look down at our profession. They don’t want to communicate with us; they don’t come down to our level, SW3.*

There are no systematic approaches that would favor a partnership between social and health care sectors on the providers’ level. Social care providers indicated that individual motivation for partnership from the medical professionals’ side is low, and representatives of both sectors indicated that there are no official pathways for intersectoral collaboration.

*We come to an agreement with the private ones [social care providers], we cooperate very well, but it is difficult with the public ones, FP7*.

*They [social workers] are hard to find because they are working only in the eldership. It is not clear where exactly to call, under what circumstances it is acceptable to call, FP11*.

#### Relationships with informal caregivers

Informal caregivers were perceived as the central figure in care organization and provision by all the participants. Health and social care providers expressed 3 types of reactions toward informal caregivers(the patient’s daughter in the vignette situation): *pressure, empathy, and guidance.* All the participants underlined that the responsibility for control of the situation lies with the patient’s daughter. They called it “*the constitutional duty*”(PPs) of the daughter to care for her parents and intended to push her “*to seek solutions*” (GPs).

*I prescribe medication and practically nothing more. Explain to the relatives about nursing home and additional care requirements for nursing identification. I will also explain that it is their constitutional duty to take care of their parents. I have my own grandmother who is 96 years old; we have hired help. We tried to care for her ourselves, went crazy after a week, and looked for another way, PP12.*

However, some respondents expressed a more empathetic attitude toward the informal caregiver, questioning her ability to take full responsibility for the care of her parents, stressing the need to share her perspective on the situation, and to pay attention to her personal expectations and her own needs for assistance.

*Relatives also need time. All information needs to be gathered and all options considered by the relatives. Maybe there would be those who want to help. It would be good for them [patients] to live in their own home, they have their own feelings and their own experiences, who knows if they would let a stranger into the house [. . .], PN8.*

The participants seldom pointed out the guiding information that could increase the informal caregiver’s general awareness about care organization (i.e., information on where to seek assistance—CNs), or shared their competence in specific care aspects (i.e., fall prevention—GPs).

#### Personal approach to LTC provision

The participants perceived the vignette situation of 2 older people with dementia as an overwhelming challenge triggering feeling of helplessness and frustration regarding the lack of professional possibilities for assistance, yet a responsible willingness to help.

*I can’t say anything good about this situation. You are powerless, PP12.*

*Since I’ve already come, I’d probably fix that ulcer. I don’t know what I can do anymore,CN2.*

*The situation is clearly impossible to solve, because I could give all my money and that would not be enough [. . .], SW10.*

Although not mentioned by the participants, it seems that these feelings may be partly due to the lack of professional cooperation in the provision of LTC in reality. When discussing the patient care options described in the vignette, they tend to rely on their personal, professional experience without a broader perspective on collaborative activities. The feeling of being left alone at a professional level can lead to professional despair and exacerbate avoidance behaviors, which can have detrimental effects on patients and their informal caregivers.

## Discussion

This study revealed challenges faced by health care and social care representatives providing services for older people with dementia in Lithuania. The main findings of our work suggest that scarcity of funding is not the sole explanation of inadequately addressed LTC needs of older people with dementia in Lithuania. Bureaucratic formalities limiting the opportunities for the provision of LTC services until the additional care requirements are determined, a deep reliance on medical and especially on institutional care, and a lack of partnership within and between the sectors result in the helplessness of formal providers and increase the pressure on the informal caregivers.

These results are consistent with previous findings that the provision of LTC in Lithuania is heavily placed on the medical sector.^23^ The participants argued that formal endorsement for LTC needs is based on medical criteria and that the endorsement procedure could solely be initiated by the health care professionals. Moreover, medical care, including in-patient care, is the only LTC possibility for patients without formal endorsement of additional care requirements. This could explain why the healthcare costs for dementia in Eastern European countries exceed social care costs, while the global pattern is the complete opposite, that is, social care costs are at least twice as high as the medical costs.^5,23^ Thus, the concerted push expressed by healthcare and social care service providers toward informal caregivers to take the full responsibility for the management of the situation revealed in our study could serve as a characteristic illustration of informal care overload in the context of low social spending on LTC. On the other hand, the specific social and cultural approach of Eastern European countries cannot be ignored when discussing the transfer of responsibilities to informal caregivers. Furthermore, LTC spending share of family responsibilities between children and their parents in Latvia are enshrined in law, meanwhile in Hungary and Lithuania—even in the constitution.^21^”

Complex solutions must be discussed in searching for a way out from this tense situation. Increasing social spending on LTC needs should perhaps be the top priority of any political manifesto. The responsibility of state and family toward LTC should be better balanced. Further, home care development to meet both medical and non-medical needs of the older people should be emphasized. Decreasing bureaucratic requirements, expansion of the spectrum of LTC services, tailoring the format of the visits to the needs of the patients, and expanding service delivery with a selfcare component^32,33^ are a few changes that could be made for a more sensitive response to the LTC needs and a more balanced distribution of the LTC burden. Well-developed home care is proven to have positive effect on lowering re-hospitalization rates, thus, diminishing medical care costs.^32^ The recent legislative amendments by the Ministry of Health, considerably enlarging the home care “gates” for all the people who are in need of LTC services regardless of the presence/absence of a formal endorsement for LTC needs, could be a step forward in this direction.^34^ These developments could have a positive impact on finding a balance between home care and institutional care in Lithuania.

A majority of the participants in our study discussed the opportunity of institutional care as the singular solution for older patients with dementia. The national policy enabling the possibility to receive up to 104 home visits per year for all patients only in the case of a formal endorsement for additional care requirements,^34^ but not applying this requirement for institutional nursing up to 120 days per year,^17^ embodies the intrinsic Lithuanian reliance on institutional care. Research indicates that Eastern and Southern European countries often follow this pattern, while Nordic and some Continental European countries put much more emphasis on community and home-based care.^21^ However, prioritization of home care over residential care should go in line with the establishment of sufficient home-based LTC services.^21^

Partnership between healthcare and social service providers is proven to have a positive impact on user satisfaction as well as leading to a more efficient use of the resources.^13,35,36^ Leading healthcare positions in LTC provision revealed in our study could also be exploited in a positive way. Research indicates that greater centrality of health care organizations in collaborative networks of healthcare and social care services organizations results in higher performance of these networks, expressed in the form of lower avoidable health care use and spending on older adults.^13^

However, the need for better coordination between social and health care sectors and the higher understanding of the social care system revealed in our study are not previously unknown issues.^37,38^ Our data suggest that the attitude of non-cooperation between social and health care providers, and a lack of systematic approaches for efficient collaboration (e.g., uncertainties about professional roles and functions, lack of mutual formal communication pathways) hamper provisions of integrated care for patients with complex needs. Previous research indicates that a policy shift toward integration is not an easily achievable task, as individual innovators, and not legal imperatives are often the key drivers of change.^8^ A study assessing the different types of collaborative ties between healthcare and social care services organizations concluded that co-sponsoring projects could be listed among the most effective ways of fostering effective partnerships.^13^ Finally, the importance of social environment and community involvement in LTC delivery should be emphasized. Research indicates that partnerships should overstep the boundaries of formal health care and social service organizations; higher performing communities also have strong informal support networks, partnerships with faith-based organizations, grassroots organizations, and advocacy efforts.^39^

## Limitations

The study was conducted with professionals working in a highly urbanized setting; the experiences of professionals from rural areas may differ. Another limitation of the study could be related to the participants’ representations where healthcare sector outweighed the social services. We suggest that further studies on the subject should utilize triangulation research wherein interview data are complemented by objective observational data.

One more limitation of our research may be that some of the interviews were not audio recorded. All participants of the study were asked to be audio recorded during the interview. However, regardless of full confidentiality assurance, some participants refused to be audio recorded. As the study itself directly reflected participants’ work, we believe that participants could some-how perceive the interviews as an audit of their work. The study results revealed the feelings of helplessness and frustration of professionals facing challenges related to long term care of dementia patients. We believe that these feelings might trigger the fear of looking nonprofessional and were the main reason participants preferred interview rather than an audio recording. As we aimed for a wide range of participants’ experiences, we decided to apply both audio-recorded and handwritten data collection techniques, always giving priority for audio-recorded interviews. As our researchers were trained to perform both audio-recorded and handwritten data collection techniques, we are confident in the quality of the data collected.

## Conclusions

The present study revealed the potential factors that could have a positive impact on the caregiving process and possibly decrease the perceived difficulties of formal caregivers. Comprehensive measures summarized in following 3 points should be addressed when developing an LTC improvement strategy. First, facilitating the endorsement of additional care requirements should be a priority and should not be based solely on the patient’s medical condition, but rather on their functional abilities. Second, improvements should focus on the establishment of clear procedures for formal cooperation between the health care and social care sectors in the trajectory of LTC service provision, with a higher awareness of the functions and roles of the representatives of both the sectors. Finally, strengthening LTC provision should focus on a more adequate balance between home care and institutional care by creating a wider range of LTC services. A more consistent and coordinated delivery of services by both the health care and social care sectors seems to be an untapped resource for the improvement of the LTC potential.
